# The Prevalence of Hepatitis E in a Patient Cohort Presenting With Addictive Injection Behavior

**DOI:** 10.3389/fpsyt.2019.00832

**Published:** 2019-11-12

**Authors:** Antoine Yrondi, Juliette Salles, Jean Marie Péron, Marie Sporer, Simon Taib, Adeline Gallini, Chloé Noilhan, Chloé Dimeglio, Flora Entajan, Marie Crequy, Jacques Izopet, Laurent Schmitt

**Affiliations:** ^1^Service de Psychiatrie et de Psychologie Médicale, Centre Expert Dépression Résistante FondaMental, CHU Toulouse, Hospital Purpan, ToNIC, Toulouse NeuroImaging Center, Université de Toulouse, Inserm, UPS, Toulouse, France; ^2^Service de Psychiatrie et de Psychologie Médicale, CHU de Toulouse, Hospital Purpan, Toulouse, France; ^3^CSAPA Maurice Dide, CHU Toulouse, Toulouse, France; ^4^Service d'Hépatologie, Hôpital Rangueil Centre Hospitalier Universitaire de Toulouse, Université Paul Sabatier Toulouse III, Toulouse, France; ^5^Service de Psychiatrie et de Psychologie Médicale, CHU Toulouse, Hospital Purpan, ToNIC, Toulouse NeuroImaging Center, Université de Toulouse, Inserm, UPS, Toulouse, France; ^6^UMR1027, INSERM University of Toulouse, Toulouse, France; ^7^Department of Epidemiology and Public Health, Centre Hospitalier Universitaire Toulouse (University Hospital Centre), Toulouse, France; ^8^INSERM U1043—CNRS UMR5282—Toulouse University Paul Sabatier, CPTP, Toulouse, France; ^9^CHU de Toulouse, Hôpital Purpan, Laboratoire de Virologie, Toulouse, France

**Keywords:** addiction, hepatitis E, injection, intravenous, seroprevalence

## Abstract

**Introduction:** Hepatitis E is the most common cause of acute viral hepatitis worldwide. Seroprevalence is approximately 15% in developed countries, and 22% in France. hepatitis E virus (HEV) can be transmitted *via* transfusions and therefore possibly intravenous (IV) drug use. Hepatitis E serology is routinely tested in patients who seek medical advice for addictive injection behavior at the addiction treatment, support and prevention unit of Toulouse University Hospital. We assume that hepatitis E is more prevalent in patients presenting with addictive injection behavior than in the general French population.

**Methods:** Hepatitis E serological assays [immunoglobulin M (IgM) and IgG] were carried out for all patients presenting with addictive injection behavior during an initial evaluation. The controls were taken from a cohort of 3,353 blood donors living in southern France and who donated blood during the first 2 weeks of October 2011.

**Results:** We included 52 patients presenting with addictive injection behavior and 103 healthy controls matched for age, sex, and area of residence. We found no difference between patients and controls for the prevalence of hepatitis E: patients vs. healthy controls: positive IgGs: 42.31%, 95% confidence interval (CI) (28.73–56.80%) vs. 43.43%, 95% CI (33.50–53.77%) (p = 0.89) and positive IgMs: 3.85%, 95% CI (0.47–13.22%) vs. 4.85%, 95% CI (0.16–10.97%) (p = 0.57).

**Conclusion:** There was no difference in HEV seroprevalence between IV drug users and the general population, suggesting that the IV route of HEV infection is not significant in this population.

## Introduction

Viral hepatitis is, unfortunately, frequently associated with addictive injection behavior (1). Hepatitis C virus is likely the most prevalent type of hepatitis among patients with addictive behavior (2), but it is not the only one. For example, there is a high prevalence of hepatitis A in this population (1). However, hepatitis E is probably the most common cause of viral hepatitis worldwide (3, 4). Seroprevalence is approximately 20% in developed countries (5) and its incidence ranges from 0.2 to 0.7% (6, 7). Hepatitis E is a virus which was discovered in Afghanistan in 1983(8). Four major genotypes currently exist [hepatitis E virus 1 (HEV-1), HEV-2, HEV-3, HEV-4] (9). Genotypes 1 and 2 are mostly found in Asia and Africa, and are transmitted by oral–fecal contamination (10). Genotypes 3 and 4 are endemic in developed countries and are transmitted zoonotically. Therefore HEV can be contracted from animals, through food or simple contact (3, 4).

The clinical symptoms of genotype 3 and 4 hepatitis E-mediated infection are similar to those of genotypes 1 and 2. Symptoms include nausea, vomiting, abdominal pain, and hepatomegaly (11, 12). Jaundice is present in 75% of cases affected by these genotypes (11) compared to 40% for genotypes 1 and 2 (12). In some cases, HEV induces neurologic symptoms (13) such as neuralgic amyotrophy, Guillain–Barré syndrome, and meningoencephalitis (14). Elevated liver enzymes is the main biological feature (11).

In developing countries, genotypes 1 and 2 are transmitted *via* the oral–fecal route (15, 16). In developed countries, genotypes 3 and 4 are mainly transmitted by animals (17, 18). The virus is either transmitted through the consumption of inadequately cooked meat (19, 20) or by simple contact (21). Contamination can occur through waste water (22, 23). Importantly, there is also a risk of transmission *via* blood transfusion (24, 25).

Diagnosis is confirmed by carrying out an anti-HEV antibody assay. Following an incubation period of two to six weeks, a short immunoglobulin M (IgM) response is followed by a longer IgG response (26). Viremia occurs in a positive IgM context (26).

Ribavirin as single therapy can be used to treat severe, acute hepatitis E (27, 28) or acute-on-chronic liver failure.

Given the fact that the virus can be transmitted *via* transfusions and that an established, effective treatment for severe forms of the disease is available, hepatitis E serology is routinely tested in patients who seek medical advice for addictive injection behavior at the addiction treatment, support and prevention unit of Toulouse University Hospital.

We assume that hepatitis E is more prevalent in patients presenting with addictive injection behavior than in the general French population.

We carried out investigations to assess whether the seroprevalence of hepatitis E was significantly higher in this target population than in the general population.

## Methods

### Population

The study is a prospective study (collection of results from the first assessment of new patients). The data are encoded. Only patients presenting at least one type of addictive injection behavior were enrolled in the study: indeed, all patients with intravenous injection are included in the study, regardless of the drug used. Due to the lack of a supervised drug consumption site, we could not know whether a safe mode was used for the injection.

We therefore matched each case (subject with addictive behavior enrolled in the ETOX study) with two controls (subjects from the general population) according to their age, gender, and area of residence. We work on the principle that, if there is a significant difference in seroprevalence between the two populations, it will not, therefore, be due to factors generally identified in relation to the patients' HEV status i.e. typically age and gender, and we consider the typical dietary habits of the area of residence (29). This difference could also therefore be explained by different practices (in this case, practices related to substance abuse/addiction).

The controls were taken from a cohort of 3,353 blood donors living in Southern France and who donated blood during the first two weeks of October 2011. All of the donors completed a lifestyle questionnaire (30). The patients were informed about the study (written and verbal information).

Hepatitis E serological assays (IgM and IgG) were carried out for all patients presenting with addictive injection behavior during an initial evaluation which also included serological tests for hepatitis B and C as well as human immunodeficiency virus (HIV). All serum specimens from patients and controls were tested for anti-HEV IgG and IgM using the Wantai HEV IgG immunoassay and Wantai HEV IgM immunoassay kits (Wantai Biologic Pharmacy Enterprise, Beijing, People's Republic of China)(29, 30).

The project was validated by the Comité Consultatif sur le Traitement de l'Information en matière de Recherche dans le domaine de la Santé (CCTIRS; French Advisory Committee for Data Processing in Health Research) and the Commission Nationale de L'informatique et des Libertés (CNIL; French Data Protection Authority) (MR003 undertaking signed by the University Hospital). All participants provided written informed consent for participation.

### Statistical Analyses

The subjects' characteristics were described using numbers and percentages for the qualitative variables and appropriate distribution parameters for quantitative variables (mean and standard deviation or median and interquartile intervals).

We estimated the prevalence of hepatitis E with its 95% confidence interval according to the exact binomial method.

The subjects' characteristics were compared according to the hepatitis E IgG serology result (2 classes—positive or negative) using chi-square tests (after checking the conditions for application: the theoretical numbers being >5) or Fisher's exact test for qualitative variables. A Student test was carried out to compare the hepatitis E IgG serology result against age after checking the conditions governing application (normal age distribution and homogeneity of variances).

The patients' characteristics are described using figures and percentages (quantitative variables).

Population-related seroprevalences were compared using chi-square tests (after checking the conditions governing application: the theoretical numbers being >5) or Fisher's exact test if the conditions for applying the chi-square test were not checked.

Analyses were performed using STATA/SE 14.2 software.

## Results

We included 52 patients presenting with addictive injection behavior and 103 healthy controls. The mean age of the patients' population was 41.63 (SD: 8.87), and there were 38 males (73.08%) and 14 females (26.92%). Twelve patients (23.08%) presented with addictive injection behavior with heroine, 12 (23.08%) with cocaine, four (7.69%) with opioid replacement treatments, 22 (42.31%) presented with multiple addictions, and two (3.85%) were addicted to other substances. Eighteen patients (34.62%) had no related oral addiction. Fifty patients (96.15%) had not travelled recently (outside France), only one had been to Africa and one had travelled outside Africa and Asia. Among the patient group, 29 (55.77%) had no history of viral hepatitis B and/or C, 17 (32.69%) had a history of hepatitis C infection, one had (1.92%) a history of hepatitis B, and five (9.62%) had a history of both. No patients presented with a concomitant HIV infection (three refused to undergo serology testing).

In this study, the prevalence of anti-HEV IgG was 42.31%, 95% CI (28.73–56.80%) and the prevalence of anti-HEV IgM was 3.85%, 95% CI (0.47–13.22%) in the group of patients displaying addictive injection behavior.

The prevalence of anti-HEV IgG and IgM in the control group was 43.43%, 95% CI (33.50–53.77%) and 4.85%, 95% CI (0.16–10.97%), respectively (**Table 1**).

**Table 1:** Serology descriptions according to patient group.

	Patient Group						
	Injection Addiction	Injection Addiction	General population	Generalpopulation	Total	Total	p value
	N	%	N	%	N	%	
**IgG Status**							0.89
Negative	30	57.69	56	56.57	86	56.95	
Positive	22	42.31	43	43.43	65	43.05	
**IgM Status**							0.57
Negative	50	96.15	98	95.15	148	95.48	
Positive	2	3.85	5	4.85	7	4.52	

The patient who had recently travelled to Africa had a positive IgG serological status.

There was no statistically significant difference in terms of positive IgG serological status between the groups with related oral addictions [12 (54.55%)] and the group without [10 (45.45%)] (p = 0.159). Similarly, there is no statistically significant difference in terms of this serological status between the group with multiple injection addictions [7 (31.82%)] and the group with a single addiction [15 (68.18%)] (p = 0.190). Finally, we did not record any difference between patients with hepatitis B and/or C viral infection [8 patients (36.36%)] and the group without any related infection [14 patients (63.64%)] amongst patients with hepatitis E positive IgG serology (p = 0.830).

None of the patients presented clinical symptoms of hepatitis E infection but two patients had evidence of recent HEV infection (positive anti-HEV IgM and negative HEV RNA).

## Discussion

The seroprevalence of hepatitis E in a patient cohort presenting addictive injection behavior does not differ from that of the general population. We found a prevalence of over 40% in our population. This very high prevalence reflects the endemic nature of this infection in southern France(3, 4). We recorded a higher seroprevalence than in studies investigating similar populations. In a Californian patient cohort presenting with addictive injection behavior, Mahajan et al. recorded a seroprevalence of 2.7% (31). Christensen et al. highlighted a seroprevalence of 24.8% in Denmark (32). Furthermore, they did not observe any differences between the group of patients with intravenous injections and the addiction group without injection behavior.

Given the high incidence, it seems essential to routinely test for hepatitis E infections in this at-risk population. Moreover, comorbidities between addiction and psychiatric disorders are common (33). There appears to be a higher prevalence of hepatitis E in patients with psychiatric disorders. Cong et al. (34) recorded a seroprevalence of 27.19% and Xue et al. (35) a seroprevalence of 34.9% in one population in China taking overall psychiatric disorders into consideration in the first study and schizophrenia in the second.

Chronic forms of hepatitis E also seem to be more common with few symptoms in patients also infected with HIV (genotype 3) (36). Although this was not found in our population, addictive injection behavior remains a risk factor for HIV infection (37).

Given (i) the potential severity of clinical signs during HEV infection, especially neurological symptoms, (13), such as meningoencephalitis or Guillain–Barré syndrome (14), (ii) the frequent comorbidities between addiction and psychiatric disorders and psychiatric disorders and hepatitis E infections (34, 35), (iii) the possibility of transmission *via* blood transfusions (24, 25), and (iv) the availability of an effective treatment, namely Ribavirin, as single therapy (27), it might be interesting to routinely propose hepatitis E serology testing in patients presenting addictive injection behavior and treated in a health care establishment. The main limit of our study is the small sample of our patient cohort presenting addictive injection behavior. This may reduce the statistical power of our study. Moreover, given that Southern France is an endemic area for hepatitis E infections, the results cannot be generalized to all parts of the world.

Further studies with a large number of patients taking psychiatric conditions, type of addiction and viral comorbidities into account would be required in order to corroborate our findings.

## Conclusion

There was no difference in HEV seroprevalence between the IV drug users and the general population, suggesting that the IV route of HEV infection is not significant in this population. However, we believe that the high seroprevalence of IgG in our region warrants routine testing in a more vulnerable population with a probably higher frequency of pre-existing hepatopathy (hepatitis B and/or C, alcohol, etc.) than in the general population: a situation that can potentially cause severe forms of hepatitis E.

## Data Availability Statement

The datasets generated for this study are available on request to the corresponding author.

## Ethics Statement

All participants provided written informed consent for participation. This study was carried out in accordance with the recommendations of the CCTIRS (French Advisory Committee for Data Processing in Health Research) and the CNIL (French Data Protection Authority) (MR003 undertaking signed by the CHU—University Hospital). The protocol was approved by the CCTIRS (French Advisory Committee for Data Processing in Health Research) and the CNIL (French Data Protection Authority) (MR003 undertaking signed by the CHU—University Hospital).

## Author Contributions

AY, JS, JP, JI, and LS: designed the manuscript. AG, CN, and CD: performed analyses and made the figures/tables. AY, JS, JP, MS, ST, MC, FE, JI, and LS: contributed to manuscript design and writing. AY, JS, MS, ST, MC, and FE: assisted with study design and were responsible for data collection. JI: analyses of anti-HEV IgG and IgM.

## Conflict of Interest

The authors declare that the research was conducted in the absence of any commercial or financial relationships that could be construed as a potential conflict of interest.
